# Practical Use of Immortalized Cells in Medicine: Current Advances and Future Perspectives

**DOI:** 10.3390/ijms241612716

**Published:** 2023-08-12

**Authors:** Nikita Voloshin, Pyotr Tyurin-Kuzmin, Maxim Karagyaur, Zhanna Akopyan, Konstantin Kulebyakin

**Affiliations:** 1 Faculty of Medicine, Lomonosov Moscow State University, 119991 Moscow, Russia; nik.voloshin.98@mail.ru (N.V.); tyurinkuzmin.p@gmail.com (P.T.-K.); darth_max@mail.ru (M.K.); 2 Medical Research and Education Center, Lomonosov Moscow State University, 119234 Moscow, Russia; zhanna.fbm@gmail.com

**Keywords:** immortalized cells, tissue engineering, regenerative medicine

## Abstract

In modern science, immortalized cells are not only a convenient tool in fundamental research, but they are also increasingly used in practical medicine. This happens due to their advantages compared to the primary cells, such as the possibility to produce larger amounts of cells and to use them for longer periods of time, the convenience of genetic modification, the absence of donor-to-donor variability when comparing the results of different experiments, etc. On the other hand, immortalization comes with drawbacks: possibilities of malignant transformation and/or major phenotype change due to genetic modification itself or upon long-term cultivation appear. At first glance, such issues are huge hurdles in the way of immortalized cells translation into medicine. However, there are certain ways to overcome such barriers that we describe in this review. We determined four major areas of usage of immortalized cells for practical medicinal purposes, and each has its own means to negate the drawbacks associated with immortalization. Moreover, here we describe specific fields of application of immortalized cells in which these problems are of much lesser concern, for example, in some cases where the possibility of malignant growth is not there at all. In general, we can conclude that immortalized cells have their niches in certain areas of practical medicine where they can successfully compete with other therapeutic approaches, and more preclinical and clinical trials with them should be expected.

## 1. Introduction

Regenerative medicine is a new field of medical science that combines the achievements of cell biology, tissue engineering, and gene therapy to achieve one goal: to ensure the restoration of damaged tissues and organs via the body’s own regenerative abilities. The key object of regenerative medicine is human cells, which act as the targets of therapy, objects of tissue engineering, and deliverers of therapeutic agents to the body, etc. This fact makes the problem of stable production of large volumes of human cells for biomedical research and therapeutic manipulations especially urgent.

The main source of cells for the needs of regenerative medicine is donor tissue; using standardized protocols, the required amounts of various kinds of cells are extracted from donor material and cultivated [1,2]. However, using material directly taken from donors has several disadvantages. One of them is cellular aging, a process in which, after a certain number of divisions, the properties of cells change, which limits the cultivation of cells from a single donor in large quantities and their widespread and long-term use [3].

Another problem with using primary donor material as a source of stem cells is heterogeneity, i.e., the difference in the properties of different cell cultures of the same type. For example, for mesenchymal stem cells (MSCs), a difference in the properties of cells from different donors, different tissues, or even when using different protocols of cell isolation, storage, thawing, and cultivation was demonstrated [4,5,6]. The heterogeneity of cells limits the comparison of the results of experiments and clinical trials not only in different institutions but even within the same laboratory [6]. One solution to these problems is the use of immortalized cell lines.

Immortalized cell lines are cell lines in which the Hayflick limit has been overcome with the help of genetic modifications, and as a result, a theoretically infinite number of divisions is possible [7]. The Hayflick limit is the limit on the number of divisions of somatic cells past which they lose their ability to proliferate; for most cells, this limit is around 52 divisions. The existence of the Hayflick limit is primarily associated with the existence of telomeres at the ends of chromosomes in cells, which shorten with each subsequent division. When the telomeres are fully shortened, any further proliferation is impossible [8,9].

Immortalization is the process of artificially overcoming the limit of cell divisions, which is carried out by changing the expression profile of the genes responsible for cell aging. The first experiments on artificial immortalization of cells were conducted in the 1960s—using polyomaviruses [10] and chemical carcinogens [11], immortalized cell lines of rats and hamsters were obtained. Currently, there are many methods of cell immortalization, including the introduction of viral oncogenes into the cell (SV40 T-antigen (Simian Vacuolating virus), HPV16/18 (Human Papillomavirus) proteins E6 and E7, and others); the introduction of the TERT gene, which is responsible for encoding the catalytic subunit of telomeras—an enzyme that builds up telomeres; and changing the expression levels of certain transcription factors (c-MYC, BMI1, ZNF217, beta-catenin, etc.; see Table 1) [7].

Reversibly immortalized cells are becoming increasingly popular. Reversible immortalization allows selective activation of the immortalizing agent, which allows for cells to be grown in the required quantities and, after the effect of the immortalizing agent is stopped, to be used without the side effects associated with the activity of such an agent. Methods of reversible immortalization include activation of the immortalization factor by adding growth factors or small molecules, using the Cre/LoxP system, and changing temperature (see Table 1) [12].

**Table 1:** Common methods of immortalization.

Immortalizing Agent	Brief Description	References
Irreversible Immortalization		
hTERT—catalytic subunit of telomerase	Ectopic expression of the hTERT gene and an increase in telomerase activity lead to the length of telomeres being maintained throughout multiple cell divisions, which prevents cell aging.	[7,13]
SV40 virus proteins (T-antigen)	Acts through many mechanisms, the main one is the binding of T-antigen SV40 with the tumor suppressor proteins (p53, Rb protein group, etc.) and reducing their function.	[7,14]
HPV16 virus proteins (E6, E7)	E6 and E7 act through a variety of mechanisms: they reduce the activity of tumor suppressor proteins (p53, Rb protein group, etc.), increase the activity of telomerase and more.	[15,16]
Transcription factor c-Myc or its viral equivalent v-Myc	Myc proteins increase the expression of hTERT, cell cycle proteins, reduce the expression of cell cycle inhibitors, etc.	[7,17]
Conditional (Reversible) immortalization		
c-MycER^TAM^	The modified c-Myc protein is only active in the presence of 4-hydroxy tamoxifen; its absence in the environment leads to a return to a non-immortalized state.	[12,18]
Tat/Dox-induced systems (HPV16 E6/E7, c-Myc, hTERT)	Various transcription initiation systems involving tetracycline (Tet-Off, Tet-On) or doxycycline led to immortalization not occurring in the absence of these antibiotics.	[12,19,20]
Temperature-controlled mutated T-antigen of SV40 (TsA58)	The mutated T-antigen is active only at temperatures of 33–34 °C; at a temperature of 37 °C, the cells return to their original state.	[12,21]
Cre/LoxP-controlled systems (for example, SV40 proteins)	Expression of the LoxP-flanked gene of the immortalizing agent can be stopped at any time by increasing the activity of Cre recombinase, which leads to cells returning to a non-immortalized state.	[12,22,23]

Various lines of immortalized cells are increasingly used both in fundamental research and in practical medicine. This review presents studies in which immortalized cells are considered a potential target for therapeutic purposes, as well as their advantages, obvious disadvantages, and potential disadvantages compared to primary cell cultures. We aimed to summarize the most prominent examples of the usage of immortalized cells for practical medicinal purposes. We used the following search strategy: First, we searched the PubMed database with the queries “Immortalized cells in therapy”, “Immortalized cells in medical practice”, “Medical use of Immortalized cells”, and “Preclinical trials of Immortalized cells” with a filter for the last 5 years. The search returned 3382 results, from which papers regarding practical medical usage were selected. Cell lines and reports were cross-referenced using the clinicaltrials.gov database.

## 2. Specifics of Immortalized Cells as an Object of Biomedical Research

Immortalization is a multistage process in which cells go through various selection options after being transfected with the necessary genetic material [24]. These manipulations can change the properties of the resulting line in comparison with the primary culture, which can limit its use not only in practical medicine, but also in fundamental research. Some possible consequences of immortalization are considered in the review by Maqsood et al. [25]: immortalization can lead to changes in the phenotype of individual cells and the properties of the entire population, for example, the cells can acquire a malignant tumor phenotype, which imposes serious restrictions on the use of such cells for the purpose of medical practice. Indeed, in all cell models, immortalization is achieved by increasing the activity of proteins associated with oncogenesis; oncogenic activity was demonstrated for the hTERT protein [26], Myc family proteins [27], HPV E6 and E7 proteins [28], and SV40 virus antigens [29].

The first publications on changes in cell properties after immortalization appeared in the early 2000s. Okamoto et al. demonstrated significant heterogeneity among MSC clones that underwent immortalization with hTERT and HPV16 E6/E7 proteins. Seven different phenotypes regarding differentiation potential were found, and only 2 clones out of 100 had differentiation potential in all three classical directions for MSCs, while 66 out of 100 did not differentiate in any direction [30]. Similar data on the differentiation potential of immortalized MSCs with the same genes was demonstrated by Takeuchi et al. [31]. 

Our laboratory obtained data confirming the results of the Okamoto and Takeuchi groups: a common commercially available hTERT-immortalized culture of ASC52Telo MSCs, with preserved osteogenic and chondrogenic potential, demonstrated a significantly reduced adipogenic differentiation potential compared to the primary culture of MSCs, and this effect is based on a decrease in insulin sensitivity due to basal Akt hyperphosphorylation [32]. Another study performed in our laboratory demonstrated a significant impairment of norepinephrine sensitivity in the same cell line [33]. 

In addition to changes in the differentiation potential of immortalized MSCs, Takeuchi et al. also demonstrated chromosomal abnormalities that occur with cells at late passages. While cells immortalized only with hTERT remained diploid after 133 doubling cycles, many tetraploid and intermediate cells appeared in the culture immortalized with hTERT and HPV E6/E7 during long-term cultivation [31]. At the same time, it is known that chromosomal abnormalities can significantly affect the properties of cells [34].

**Figure 1:**
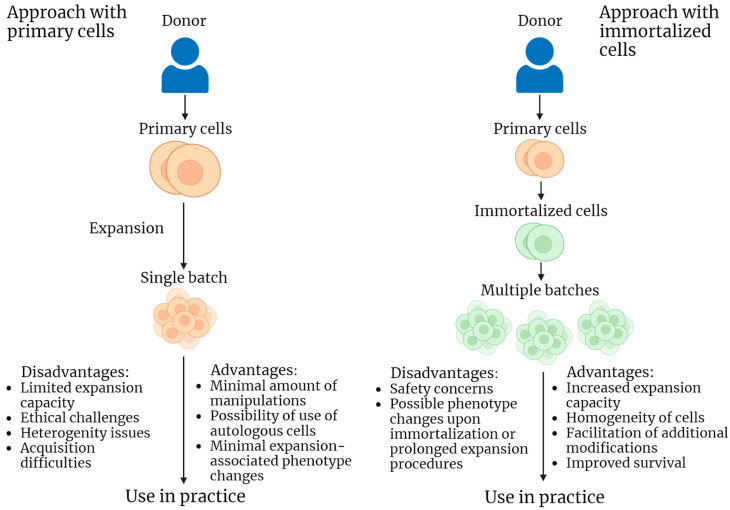
Schematic representation of two strategies of cellular product acquisition and application for practical purposes: use of primary cells and use of immortalized cells. Advantages and disadvantages are listed for each strategy.

It is apparent that immortalized cell lines may differ in properties from primary cell cultures of the same type. The main concern is the possible malignant transformation of the immortalized cells because of an increase in the activity of genes that promote oncogenesis (all possible drawbacks and benefits of immortalized cell lines are presented on Figure 1). A prospective solution to such problems is a more detailed examination of the obtained immortalized lines in comparison with primary cell cultures. As presented below, many authors in their studies on the use of immortalized cells in practical medicine pay special attention to the problems of matching the properties of immortalized and primary cultures, as well as the oncogenic potential of the studied lines. At the same time, these issues are not so acute in some areas of the application of immortalized cells.

## 3. Immortalized Cells in Regeneration

The main area of application for cellular products is regenerative medicine. Many cell cultures are actively studied and used for regenerative purposes in various diseases [35]. Due to more comfortable working conditions and higher survival and proliferative potential, some researchers are trying to use immortalized cell lines for regeneration purposes.

### 3.1. CTX0E03

One of the most popular immortalized cell lines currently used for medical purposes is the reversibly immortalized human fetal neural stem cell line CTX0E03 obtained in 2006 by Pollock et al. [36]. This line was obtained using the 4-hydroxy tamoxifen-dependent transcription factor c-Myc (c-MycER^TAM^ technology). In the original study in 2006, the authors demonstrated the ability of these cells to restore motor function in mice after a simulated cerebral stroke by directly injecting cells into the pathological focus 3–4 weeks after injury. This effect was mediated by cell differentiation in the neuronal direction, which was confirmed by histological analysis data. The authors also studied the oncogenicity of cells: in vitro and in animals, CTX0E03 cells demonstrated no oncogenic potential in contrast to irreversibly immortalized cells [36].

To date, the therapeutic efficacy of CTX0E03 cells has been evaluated in two series of clinical trials. PISCES is a series of clinical trials dedicated to the use of CTX0E03 cells to reduce the consequences of cerebral ischemic stroke when used several months after a stroke. In the first phase, safety and the absence of side effects directly related to cells injected into a pathological focus of up to 20 million cells were demonstrated, as well as some positive progress in neurological symptoms [37]. In phase IIa, a positive effect on motor functions was confirmed, though not in all patients, but only in the group that maintained residual motor functions before the start of therapy [38]. The published outcomes of the phase IIb clinical trial (PISCES III) involving 15 participants indicate that there were no observed neurological improvements in patients who received a 20 million cell injection compared to those in the placebo group. These results were evaluated over a period of 6 months following the procedure [39]. Information regarding an ongoing long-term safety study of CTX0E03 cell injection, including participants in the PISCES III trial, is also accessible [40].

There are also data on the completed first phase of the clinical trial of CTX0E03 cells for the treatment of lower limb ischemia, but no results have been published in this trial either [41].

The ability of CTX0E03 cells to survive in an in vitro model of Alzheimer’s disease was assessed by Puangmalai et al. by culturing in an environment containing various concentrations of amyloid β or okadaic acid, a protein phosphatase inhibitor that creates a high concentration of phosphorylated tau protein. The CTX0E03 cell line demonstrated similar or better survival rates compared to primary cultured rat neuronal stem cells, making them potential candidates for cell therapy in Alzheimer’s disease [42]. At the same time, the study of Yoon et al. demonstrated the effectiveness of using CTX0E03 cells for the treatment of Huntington’s disease in the quinolinic acid-lesioned mouse model [43].

Despite some issues with the methodology of clinical trials (small sample size, wide range of time elapsed since stroke, lack of female patients in phase I [44,45]), many preclinical [42,43,46] and clinical studies demonstrate that the CTX0E03 cell line is a promising therapeutic tool with a significant neuroregenerative potential, both in models of ischemic damage and in models of neurodegenerative diseases. The advantages of this line in comparison with the primary culture are obvious, such as the absence of the need for obtaining and isolating material and the standardization of research results. At the same time, there is a lack of studies directly comparing the neuroregenerative efficiency of CTX0E03 cells with that of primary cultures of neuronal stem cells. However, despite the safety demonstrated in the original study [36] and in clinical studies [37,38], there are still concerns about the possibility of a change in the phenotype and malignant transformation of cells in the long term [45]. In general, the CTX0E03 cell line is a successful example of the use of reversibly immortalized cells in practical medical applications. However, more data on their efficacy and safety are needed to expand the scope of their application.

### 3.2. Other NSC in Neuroregeneration

In a series of experiments on mice, a line of human fetal neural stem cells, HK532-IGF-1, reversibly immortalized with c-Myc and modified to increase the expression of IGF-1, was evaluated for the treatment of Alzheimer’s disease. These cells demonstrated higher survival in an in vitro model of Alzheimer’s disease compared to primary cell culture and had a positive effect on a model of Alzheimer’s disease in mice when injected into pathological foci. However, this series of studies lacks data on oncogenicity and efficacy comparisons with primary cell culture [47,48,49].

Another cell line actively used as an object with high neuroregenerative potential is the HB1.F3.BDNF line of human fetal neural stem cells irreversibly immortalized with v-Myc [50] and modified for BDNF expression. Recently, preclinical studies in mice have demonstrated the effectiveness of this line for the treatment of hemorrhagic stroke [51], Huntington’s disease [52], and in a model of spinal cord injury [53]. However, studies of this line also pay little attention to safety in terms of possible oncogenic potential, especially given the irreversibility of immortalization.

In general, immortalized cells compare favorably with primary cells in the field of neuroregeneration, primarily in terms of ease of use and comparisons of the results of different experiments. In addition, due to their better survivability, they may also have a higher therapeutic potential compared to primary cultures, which makes them especially relevant considering the pronounced lack of therapeutic agents with neuroregenerative properties. The main barrier in this area, despite the reversibility of the immortalization of most of the studied lines, is the oncogenic potential of the cells used, which, however, is considered and actively studied both in preclinical and clinical studies.

### 3.3. Immortalized Blood Cell Precursors

The prospect of obtaining an erythroid precursor is extremely attractive for solving the problem of donor blood availability. An immortalized erythroid precursor would make it possible to produce large quantities of more standardized, infection-free cells, including those of rare blood types (for example, Rh-null “Golden” blood, with a few dozens of people worldwide having it). At the same time, there is no need to be wary of the oncogenic potential of immortalization, since the ideal end product is non-nuclear cells. Therefore, several studies have been carried out to obtain an immortalized erythroid precursor.

In 2013, Kurita et al. produced two immortalized cell lines: the HiDEP line from induced pluripotent cells and the HUDEP line from cord blood cells using the Tat-induced HPV16 E6/E7 protein system. Although the efficiency of differentiation of these cells into non-nuclear erythrocytes was low and only one line (HUDEP-2) expressed 2-alpha-2-beta hemoglobin (while others expressed fetal forms of hemoglobin), this is the first study in which immortalized erythroid progenitors able to differentiate into cells comparable to primary erythrocytes in their ability to carry oxygen were obtained [54].

In the same year, Hirose et al. published data on the production of imERYPC lines, erythroid precursor cells immortalized by the Dox-induced c-Myc system, and BCL-XL derived from embryonic stem cells or induced pluripotent stem cells. However, the differentiation efficiency of these cells was low, and only 0.36% of the cells obtained were non-nucleated. Also, no lines expressing 2-alpha-2-beta hemoglobin were produced [55].

A more efficient progenitor was obtained by Trakarnsanga et al. BEL-A is a line of adult CD34+ human bone marrow cells immortalized by the Tet-induced protein system HPV16 E6/E7 that was obtained in this work. The authors demonstrated that after differentiation, up to 30% of cells do not have a nucleus, and they also synthesize exclusively 2-alpha-2-beta hemoglobin, which distinguishes them favorably from all previously described cell lines [56].

In 2022, Soboleva et al. obtained an irreversibly immortalized human bone marrow cell line, ELLU (Erythroid Line from Lund University), using HPV16 E6/E7 proteins. In this work, the authors emphasize that it is possible to obtain an effective immortalized erythroid progenitor using irreversible immortalization, while previously it was believed that a decrease in the activity of the immortalizing agent was necessary for successful differentiation of cells into non-nuclear erythrocytes. The cells of the resulting line turned out to be heterogeneous in the type of hemoglobin they express, in the rate of differentiation, and in the fragility of differentiated cells [57].

A paper by Kim et al. specifically focuses on the effectiveness of irradiation and leukoreduction filters in removing nucleated cells for manufacturing blood products from immortalized precursors. Their experimental findings suggest that these methods do not eliminate oncogenic material, indicating a need for further advancements in this area to ensure the safety of immortalized blood precursor products [58].

The 2013 paper by Trakarnsanga and co-authors also addresses the main challenges that stand in the way of translating the findings into practice. In addition to the efficiency of differentiation, the lack of an effective system for the mass production of erythroid cells from immortalized progenitors and the problem of purification of non-nucleated cells from earlier progenitors, which raise concerns in terms of oncogenicity due to the possible residual activity of the immortalizing agent, are the main barriers to the future use of these cells in medicine [56].

Another problem of modern medicine is obtaining immortalized megakaryocytes, precursors of platelets. Allogeneic platelet transfusion is actively used to treat hemorrhagic conditions in patients with thrombocytopenia, for example, in aplastic anemia; however, patients often develop an allergic reaction to platelet antigens, such as HLA-1 and HPA (Human Platelet Antigens), and if the patient has a rare set of such antigens, allogeneic platelet transfusion becomes impossible due to the lack of suitable donors. For such patients, the solution could be the creation of autogenous immortalized megakaryocytes, from which it is possible to accumulate the required number of platelets to compensate for pathological conditions.

A 2022 paper by Sugimoto et al. describes the production and use of autologous human megakaryocytes derived from induced pluripotent stem cells (in turn derived from peripheral blood mononuclear cells) and immortalized by doxycycline-induced expression of c-MYC, BMI1, and BCL-XL—imMKCL cells (immortalized MegaKaryocyte progenitor Cell Line). For the growth and differentiation of these cells, the authors used not only doxycycline, but also other substances (a thrombopoietin mimetic, an inhibitor of Rho-associated kinases, and several other factors that promote platelet maturation); a bioreactor maintaining a turbulent flow of the medium was also used, which promotes thrombopoiesis. As with the immortalized erythroid progenitor, the end product is non-nucleated and thus non-hazardous in terms of oncogenic potential; however, concerns remain about the nuclear progenitor cells that remain in the final product. The authors note that chromosomal abnormalities are observed in imMKCL cells after the 20th passage. Notably, Sugimoto et al. used 25 Gy radiation exposure to reduce the oncogenic potential of the resulting cell product; irradiated cells showed zero oncogenic potential compared to the same non-irradiated cells in vitro. The introduction of platelets derived from the imMKCL cell line (iPSC-PLT) promoted hemostasis in vivo in the rabbit [59]. 

The second study by Sugimoto et al. is devoted to a phase I clinical trial of iPSC-PLT platelets to compensate for hemorrhagic conditions in a donor of these exact cells with a rare set of platelet antigens who suffers from aplastic anemia and has developed an immune response to allogeneic platelets. To our best knowledge, this is the only autologous immortalized cell study in humans. The authors investigated the safety and efficacy of administering various doses of iPSC-PLT (after irradiation): administration of all doses was safe (observation period: 1 year) but showed zero efficacy in the CCI parameter (Corrected Count Increment) after 1 and 24 h, which is used to evaluate the effectiveness of platelet transfusion [60]. The authors attribute the demonstrated low efficiency to the possible incorrect endpoint selection—it is possible that the peak of CCI falls on the period of 2–6 h, which was demonstrated in a rabbit in a work dedicated to obtaining these cells [59]. 

**Figure 2:**
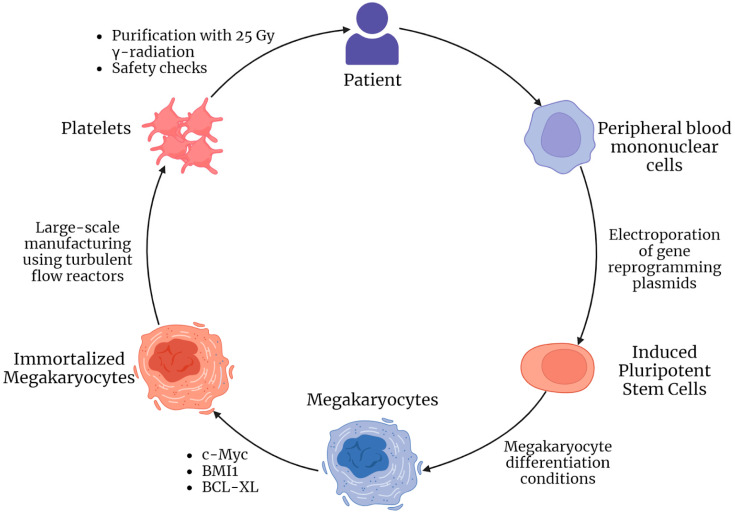
Pipeline of immortalized autologous imMKCL megakaryocytes and iPSC-PLT platelets engineering and application presented in articles by Sugimoto et al. [59,60].

In general, these studies by Sugimoto and co-authors (summarized in Figure 2) are unique in two respects. First, they demonstrated the effectiveness of radiation exposure to reduce the oncogenic potential of the final cellular product; this method may also be relevant for other applications of immortalized cells, for example, an immortalized erythroid progenitor—it is known that mature erythrocytes are also resistant to radiation [61]. Secondly, these studies demonstrated the full cycle of obtaining, manufacturing, and quality control of an autogenous immortalized cell precursor, as well as the use of its differentiation products, which testifies in favor of the relevance of cell immortalization in the development of personalized cell products.

### 3.4. Immortalized MSCs in Regenerative Medicine

MSCs are increasingly used as a therapeutic agent in various fields of medicine, such as the treatment of various diseases of bone tissue, disorders of adipose tissue metabolism, and autoimmune and oncological pathologies [62,63,64]. Several attempts were also made to study the regenerative abilities of immortalized MSCs. In 2006, Honma et al. demonstrated the positive effect of peripheral vein injection of hTERT-immortalized human bone marrow MSCs in a mouse model of ischemic stroke 12 h after injury [65]. Similar results were obtained in 2019 by Li et al.: the hTERT-immortalized human MSC line demonstrated higher efficacy in treating ischemic brain damage in rats after intravenous injection into a peripheral vein compared to the allogeneic primary culture of human MSCs [66].

In 2009, Nakahara et al. obtained a line of hTERT-immortalized human bone marrow MSCs, YKNK-12, and demonstrated the high efficiency of osteoblasts derived from these cells in the regeneration of bone tissue in mice. The authors also note the complete absence of tumors for 6 months at the sites of cell transplantation; however, they do not reject the possibility of a change in the phenotype to a malignant one during long-term cultivation [67].

In 2016, Kim et al. obtained two lines of hTERT-immortalized mouse adipose tissue MSCs named mADSCshTERT: the CD34+ line and the CD34− line. This work demonstrated the positive effect of these lines on a mouse model of acute myocardial infarction when injected into the pathological focus immediately after injury, as well as a pronounced general anti-inflammatory effect when administered systemically. It is noteworthy that in this work a comparison (using the results of previous studies) of the regenerative potential in the treatment of lower limb ischemia in a mouse model with primary cells is made, and the potential of immortalized cells turned out to be significantly lower. It has also been demonstrated that the resulting immortalized cells secrete significantly less IL-6 compared to the primary cells. The authors also addressed a possible change in the properties of the population at late passages: no differences were demonstrated in the proliferative and differentiation potential of cells after 100 population doublings [68].

In the study by Zhu et al., the imMSCs/eSDF-1+ bone marrow MSC line was obtained, reversibly immortalized with c-Myc and hTERT under a tetracycline transactivator, and modified for increased expression of SDF-1, which plays an important role in tissue repair. This line demonstrated a higher efficiency in the regeneration of the pelvic nerve after a week of injection into the affected area in a mouse model compared to allogeneic primary cell culture [69]. Chiu et al. demonstrated a positive role for MSCs from mouse bone marrow immortalized by retrovirus-mediated insertional mutagenesis (the exact method of immortalization was not specified by the authors) in the regeneration of muscle tissue in mice [70].

Thus, various immortalized MSC lines have a significant regenerative potential for various tissues, which allows us to consider them as potential candidates for application in practical medicine. The main barriers to the development of this field are the low attention paid to the oncogenic potential of cells as well as the significant competition from primary MSCs. To date, more than 1000 clinical trials with primary MSCs have been conducted, and several drugs based on them are available on the market [6,63].

## 4. Immortalized Cells as Deliverers

Targeted drug delivery is an increasingly popular area of modern oncology, the essence of which is the transportation of substances that inhibit the growth of tumor cells directly to the tumor area with some vector. For this, a wide variety of technologies are used, including living cells. Various primary lines and modified MSCs and HSCs have been used for a long time as deliverers for the therapy of oncological diseases due to their homing abilities [71]. However, such technologies have a number of disadvantages, such as the problem of obtaining a large number of cells for large-scale trials and the possibility of the effectiveness of treatment decreasing due to the rapid death of delivery cells. Because of this, several studies have been dedicated to the use of immortalized cell lines for the delivery of substances to tumors, which could potentially solve these problems.

The work of Lee et al. demonstrated the production of a line of fetal MSCs from human bone marrow, irreversibly immortalized with the SV40 T-antigen and modified to express the thymidine kinase of the herpes simplex virus. When injected into the systemic circulation, such cells accumulate in sites of high vascularization, especially in tumors, and when a prodrug, in this case ganciclovir, is used, it is converted into the active substance in them, which then results in a local increase in the concentration of the cytotoxic agent. The obtained SV40-TK-hfBMSC line demonstrated high efficiency in a mouse model with implanted prostate cancer cells. This study does not compare efficacy with a similar non-immortalized cell culture. The authors note the absence of tumors that developed from the injected cells during the observation period; however, they do not completely exclude the possibility of malignant transformation of the obtained cells at late passages during cell cultivation in vitro [72].

Another series of studies is dedicated to the study of neuronal stem cells of the HB1.F3 line (described in the previous section, [50]), modified for the expression of cytosine deaminase (HB1.F3.CD line), and obtained in 2006 by Kim et al. [73]. The prodrug in this case is 5-fluorocytosine, which is converted to the cytotoxic 5-fluorouracil. This cell line and its various modifications aimed at increasing the expression of certain proteins demonstrated an antitumor effect on hepatocarcinoma cells in vitro [74], in animal models of breast cancer [75], melanoma [76], and choriocarcinoma [77].

In a phase I clinical trial, the safety of intracranial administration of HB1.F3.CD cells in patients with glioma was shown in combination with a subsequent seven-day course of 5-fluorocytosine. The overall safety, the absence of side effects associated with the cells, and the low survival of these cells in the tumor area after the end of the course of chemotherapy have been demonstrated. The authors also note the low oncogenic potential of these cells, which they attribute primarily to the fact that a high concentration of cytotoxic 5-fluorouracil is constantly present in their environment. At the same time, the effectiveness of a single injection cycle and a seven-day course of chemotherapy was low; however, it should be taken into account that the use of 5-fluorouracil is not standard for patients with glioma [78].

Data has been published from a similar phase I clinical trial by the same group of authors, investigating the safety of repeated intracranial injection of higher doses (compared to the previous clinical trial) of HB1.F3.CD cells and subsequent chemotherapy courses. In this study, higher doses resulted in more pronounced side effects in participants, while the efficacy compared to the clinical study described in the previous paragraph did not change significantly [79].

Another area of application for immortalized cells as deliverers is the problem of chronic pain. A whole series of studies is dedicated to the potential use of various immortalized and modified cells to express neuropeptides that reduce pain sensitivity. In particular, a line of rat astrocytes, IAST/GAL, irreversibly immortalized with the SV40 T-antigen and modified to express galanin, a neuropeptide with pronounced antinociceptive properties, was obtained [80,81]; it showed efficacy in the treatment of pain in vivo in a rat model of sciatic nerve injury when implanted in the subarachnoid space of the lumbar spinal cord a week after the injury [81]. A line of the same cells but modified for the Tat-on inducible expression system of preproenkephalin, a precursor of an endogenous agonist of opioid receptors, was also obtained (IAST/hPPE (human PreProEncephalin) line). These cells also showed efficacy in vivo in a similar rat model of sciatic nerve injury when injected into the subarachnoid space of the lumbar spinal cord, and the analgesic effect was significantly increased when doxycycline, an activator of preproenkephalin expression in these cells, was added to the drinking water of rats [82]. A recent advance in this field is the generation of irreversibly hTERT-immortalized bone marrow MSCs modified for inducible galanin expression using the Tat-on system, the hTERT-BMSCs/Tet-on/GAL line. These cells also showed efficacy in the same rat model of sciatic nerve injury when similarly injected into the subarachnoid space of the lumbar spinal cord, and efficacy was significantly increased when doxycycline was added to drinking water. The authors also note that MSCs are easier to obtain in comparison with astrocytes [83]. For this line, the work devoted to its production also describes the conformity of the properties of the obtained cells (before modification with the Tet-on/GAL system) to the primary culture, as well as the absence of oncogenic potential, which was evaluated in vivo for 4 months after injection into soft tissues in mice [84].

**Figure 3:**
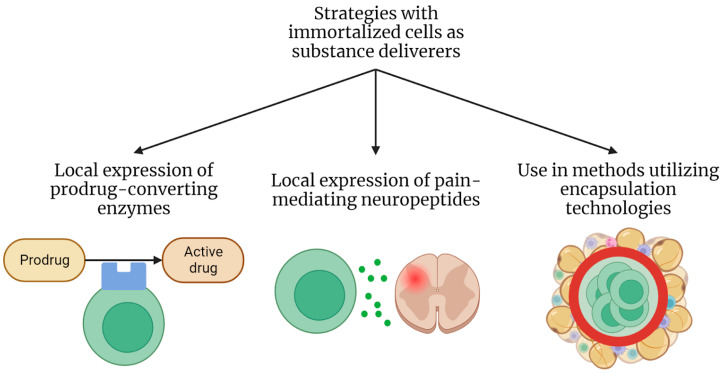
Areas of application of immortalized cells as therapeutic substance deliverers.

In modern medicine, the principle of implantation of cells as producers of various substances is being actively studied using encapsulation technology, in which cells delimited by an artificial membrane that is permeable to substances secreted by cells are introduced into the body. This approach is currently being actively developed for the treatment of diabetes mellitus (encapsulated cells that produce insulin), the intraocular delivery of various substances, the treatment of neurodegenerative diseases (introduction of encapsulated cells producing various neuroactive peptides), and other conditions (reviewed in [85]). However, not all cell lines are suitable for encapsulation: the problem of excessive cell growth manifests after the introduction, which leads to a lack of nutrients and oxygen at the center of the capsule and the formation of a necrotic nucleus, as well as the problem of cell immunogenicity due to the permeability of the capsule for the molecules of the immune system, and others. These issues are the reason why very few cells have been used as material for such studies. A recent study by Lathuiliere et al. describes the production of a human myoblast line immortalized with hTERT and CDK4 that was modified either to express GM-CSF (used in tumor therapy as a factor contributing to the development of an immune response to tumor cells) or various macromolecules—antibodies or SARS-CoV-2 spike protein—and is suitable for encapsulation. These cells have demonstrated the ability to proliferate inside the capsule of the MyoPod device in vitro, as well as good survival when this device is implanted in the tissues of an immunodeficient mouse, which indicates that there is no tendency to form a necrotic nucleus due to a lack of nutrients. The authors note that this line compares favorably in terms of immunogenicity; myoblasts themselves are less likely to elicit an immune response. These cells also cause little concern in terms of oncogenic potential since they are planned to be used only in capsules, which prevents the cells from encountering the internal body environment, and it is possible to remove the capsules at any time. The authors state that they plan to conduct a stage I clinical trial with a GM-CSF-producing variant of these cells for tumor therapy [86]. The possible areas of application of immortalized cells as substance deliverers are summarized in Figure 3.

Thus, immortalized cell lines are being actively studied and compete with primary cells and other methods in the field of targeted therapy of oncological diseases and the delivery of therapeutic substances. The main limitation of their more widespread use in oncological diseases is the low efficacy showcased in clinical trials (however, in the clinical trial of the HB1.F3.CD line, the cells converted the drug, which shows low efficacy in the chosen model; at the same time, the safety of intracranial administration of low doses of cells was demonstrated in the same trials [78]). In this area, in general, the use of immortalized cells is subject to the problems that are typical for the use of any cells as deliverers of substances: choosing the number of cells to be injected to account for possible toxicity or insufficient efficiency, the problem of distribution, and cells spreading into unwanted locations [71]. The problem of oncogenicity in this area is most relevant for delivery cells of antinociceptive molecules; it is less relevant for cells used for tumor therapy since their use implies a constant high concentration of anticancer drugs in the environment. The issue of oncogenicity is the least relevant for encapsulated delivery cells, which are delimited from the internal environment of the body by a capsule membrane and can be removed from the body at any time. It should also be considered that immortalized cells in the field of delivery of antitumor substances compete not only with primary analogues, but also with other methods of targeted therapy delivery—delivery using liposomes, extracellular vesicles, viral vectors, polymer particles, and others [71]. To overcome the barriers listed above, further studies of existing models and the development of new ones are required.

## 5. Immortalized Cells as Producers

An alternative approach to the use of cellular products in regenerative medicine is the use of stem cell secretomes as therapeutic agents. Many studies have demonstrated the effectiveness of the conditioned medium or its components of various stem cells in many pathological models. The regeneration effect is achieved through high concentrations of angiogenic factors, growth factors, various cytokines and chemokines, as well as exosomes [87,88].

Several studies have been conducted on the use of immortalized cell secretomes as a potential therapeutic agent. In 2016, a group led by Park obtained an hTERT-immortalized Sca-1+/CD31−CSCshTERT cell line, which is a modified CD31− subpopulation isolated from resident mouse heart stem cells. Although the secretome of these cells differed from the secretome of primary cells due to a decrease in the expression of VEGF and IL-6, in vitro, the effectiveness of the secretome of these cells in protecting mouse cardiomyocytes from hypoxia due to high concentrations of MCP-1, EGF, IGF-1, IGF-2, HGF R, and other factors was demonstrated [89]. Later, the same group of authors demonstrated that when forming spheroids based on poly-HEMA, these cells produce more factors involved in protection against hypoxia, and the effectiveness of the resulting spheroids in the treatment of the acute phase of myocardial infarction in vivo in mice when injected directly into the pathological focus was confirmed [90].

The work of Strenzke et al. showed a positive role for the secretome of the MSC line SC1GFP/SCX that was hTERT-immortalized and modified to express the transcription factor Scleraxis (thus mimicking the phenotype of tendon progenitor cells) in the regeneration of muscle tissue in vitro [91].

In our research, we explored the potential application of the secretome of a commercially available line of hTERT-immortalized human MSCs, ASC52Telo (ATCC^®^ SCRC-4000™), to prevent fibrosis. The secretome of these cells significantly reduced the efficiency of TGFbeta-induced differentiation of fibroblasts into myofibroblasts in vitro (which is evidence of their ability to stimulate healing without fibrosis), and it was shown that extracellular vesicles and their miRNAs play a key role in this [92].

In a 2020 study by Kraskiewicz et al., hTERT-immortalized adipose tissue MSC lines HATMSC1 and HATMSC2 were obtained from a healthy donor and from a donor with a chronic venous ulcer. The authors demonstrated the equally high regenerative potential of the conditioned medium obtained from these cells in an in vitro chronic wound model [93]. In a recent 2021 study, the same group of authors demonstrated that HATMSC line cells secrete more pro-regenerative factors than primary MSCs, and the regenerative efficiency of the supernatant of these cells in vitro was also demonstrated once more [94].

Similar results are described by Iacomi et al. in 2022: an hTERT-immortalized human ASC line S1-ADSC was obtained, the conditioned medium of which has a positive effect on keratinocyte proliferation, as shown in an in vitro 2D model, and on skin regeneration in general, which was also demonstrated in a 3D model in vitro [95]. 

Knight et al. in 2022 obtained a line of hTERT-immortalized progenitor cells of the lamina propria of the human oral mucosa called OMLP-PCL (Oral Mucosa Lamina Propri—Progenitor Cells). These cells are similar to MSCs in their properties; however, as the authors state, they have a higher regenerative potential since regeneration in the oral cavity takes place without scarring. The authors showed that these cells retained their differentiation potential compared to the same cells before immortalization, and the capacity for adipogenic differentiation in immortalized cells turned out to be even higher. Extracellular vesicles produced by the cells of the OMLP-PCL line showed a positive effect on the proliferation and migration of fibroblasts, as well as an inhibitory effect on their differentiation into myofibroblasts in vitro (which indicates their ability to stimulate healing without fibrosis), and these effects are much more pronounced than the effects of extracellular vesicles derived from primary bone marrow MSCs. The extracellular vesicles from these immortalized cells have also demonstrated a positive effect on scar-free wound healing in a mouse model [96]. 

Exosomes are receiving more and more attention as a potent therapeutic agent, and some authors see immortalized cells as a solution for the manufacturing of exosomes on a large scale. Exosomes derived from the CTX0E03 cell line described in the regeneration section were evaluated as a cardioprotective agent by Katsur et al. Exosomes derived from differentiating CTX0E03 cells showed a significant cardioprotective effect in mice when injected into the jugular vein 5 min before myocardial injury. The authors also demonstrated in vitro using the HL-1 cardiomyocyte line that the protective effect of these exosomes is associated with the activation of the gp130/JAK signaling pathway [97]. In a recent paper by Labusek et al., exosomes derived from immortalized MSCs are shown to have similar neuroprotective capacity as exosomes from primary MSCs in a mouse model of neonatal hypoxic-ischemic brain injury [98]. An article by Zhang et al. shows a positive effect of topically applied exosomes derived from E1-MYC-immortalized human MSCs in a mouse imiquimod-induced psoriasis model [99]. Recent work by the same group of authors describing the research roadmap of MSC exosomes mentions that exosomes from these cells are currently used in a phase I clinical trial regarding the topical application safety of MSC exosome ointment [100,101]. 

Since, when using a conditioned medium, the final therapeutic product does not contain cells (the concept of cell-free therapy [88]), the use of immortalized cells for this purpose does not imply any concerns associated with an altered phenotype compared to primary culture combined with the use of appropriate filtration systems. On the contrary, the possibility of longer cultivation and, consequently, the production of larger amounts of a conditioned medium favorably distinguish immortalized lines from primary cultures. Possible problems here are the changes in the spectrum of secreted factors after passing through the immortalization procedure and over numerous passages, which are solved by careful study of the secretome of candidate cells immediately after obtaining a cell line and during long passages. We believe that in this area, due to the significant advantage of immortalized cells compared to primary cells and the absence of major disadvantages, significant discoveries and rapid progress should be expected.

## 6. Immortalized Cells and Bioartificial Organs

Although the field of obtaining and producing bioartificial organs is still developing [102], some authors have evaluated the possibility of using immortalized cells for this purpose. Along with oncological concerns, it is also important to consider the immunogenicity of cells in this area; however, as presented below, in some cases of bioartificial organs, these problems are not as relevant.

In a series of publications by Mihajlovic et al., a line of ciPTEC proximal tubular cells reversibly immortalized using temperature-induced SV40 and hTERT [103] was evaluated for possible use in a bioartificial kidney (while dialysis machines only compensate for the filtration function of the kidney, the bioartificial kidney has the potential to compensate for the homeostatic, regulatory, metabolic, and endocrine functions of the kidneys [104]). This is a device with a hemodialysis function (external or implantable), some design variations of which assume that the cells of the construct will be fenced off from the internal environment of the body by a membrane, so the issue of oncogenicity and immunogenicity of the future construct is much less acute here. Despite this, in their work, the authors assessed the immunogenic potential of these cells as low [105]. In another study, the authors made a detailed evaluation of the oncogenic and metastatic potential of these cells and concluded that ciPTEC line cells do not possess any oncogenic potential and are safe for future use in bioartificial structures [106,107]. Currently, ciPTEC line cells are being studied in terms of their excretory function [108] and are also actively used in the development of bioartificial tubular constructs [109,110].

Another actively developed bioconstruct is the bioartificial liver, which, in some variants of its design, also avoids direct contact between cells and the internal environments of the body [111]. Certain variants of reversibly immortalized hepatocytes were considered for possible use in a bioartificial liver in the early 2000s [112]. In the next decade, several immortalized lines were developed that could potentially be used in the development of a bioartificial liver: immortalized with the help of SV40 antigens [113,114,115], including those dependent on temperature [116,117], HPV16 E6/E7 + hTERT [118], Cre/LoxP-associated system of reversible expression of hTERT [119], SV40 antigens [120,121] hepatocyte lines, and others (reviewed in [122]). Some of these studies have demonstrated a significant effect of transplantation of these cells in various animal models of liver failure [115,117,119,121]. In a 2020 paper, Li et al. demonstrated an Ali-BAL (Air–Liquid interactive Bioartificial Liver) bioconstruct obtained using HPV16 E6/E7 immortalized iHepLPC hepatocyte progenitor cells. This design has demonstrated efficacy in a porcine model of acute liver failure, not only in terms of maintaining and increasing the function of target organs for toxins, the concentration of which increases in liver failure, but also in terms of regeneration of the experimental animal’s own liver [123]. Currently, iHepLPC line cells are actively used in other works focused on the development of scaffolds and lobular structures for future bioartificial livers [124].

In 2015, Reijnders et al. obtained a skin model from hTERT-immortalized human keratinocytes. However, the authors suggest using such a model mostly in in vitro studies; much less attention is paid to its potential use in practical medicine [125].

Also, several works are dedicated to obtaining a model of the cornea using various lines of SV40 T-antigen-immortalized cells (MCEC—Mouse Cornea Endothelial Cells [126], ihCE-TJ—immortalized human corneal epithelial cells [127]). However, these studies are also more focused on the fundamental properties of cells and the cornea than the use of the obtained constructs in practical medicine.

Siska et al. obtained an hTERT-immortalized MSC line from Wharton’s jelly GB/hTERT MSC that produces a glucose-sensitive biosensor. The authors note that such a cell line can be used in an implantable device that will facilitate more convenient measurement of glucose levels in human blood plasma. This study also demonstrated the low immunogenicity and oncogenicity of the obtained cell line [128].

**Figure 4:**
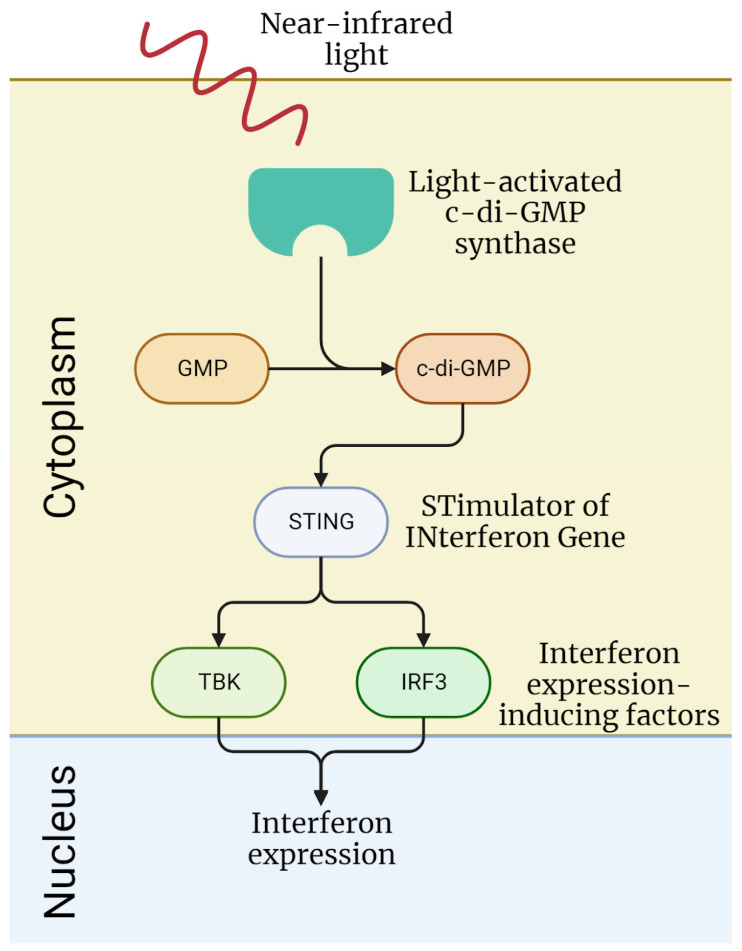
Signaling pathway of interferon expression followed by near-infrared light exposure of the hTERT-MSC Optoferon line obtained and tested in work by Audoard et al. [130].

A recent paper by Audoard et al. describes a hTERT-immortalized MSC line modified for near-infrared light-induced expression of interferon-beta for multiple sclerosis management (Optoferon line). In the original study about the engineering of these cells (hTERT-MSC line before additional modification) by Simonsen et al., the absence of chromosomal abnormalities after 96 population doublings and the absence of tumor formation for 6 months after subcutaneous implantation of these cells in immunodeficient mice were shown [129]. Audoard et al. modified these hTERT-MSC to express bacterial light-activated cyclic diguanylate monophosphate (c-di-GMP) synthase and inserted a STING construction (STimulator of INterferon Gene) that releases interferon expression-inducing factors (TBK1 and IRF3) upon binding with c-di-GMP; as a result, these cells express interferon-beta when exposed to near-infrared light, which is shown in vitro (Figure 4). Also, the engineering of a device for the implantation of these cells and the controlled interferon release is described, and a prototype of such a device was evaluated for management of multiple sclerosis in an EAE mouse model; its efficacy when implanted subcutaneously is estimated to be equal to the alternative strategy of adenoviral delivery of interferon-expressing genetic constructs [130]. In this paper, the authors also described why they chose immortalized cells for this device: they state that these cells are already validated for therapeutic use and have known properties regarding toxicity and possible tumorigenesis, and that the use of a validated cell line will facilitate the translation of a device to the clinic [130]. Overall, this work shows that immortalized cells can become the basis for the engineering of bioartificial constructs designed for controlled therapeutic substance expression.

This suggests that immortalized cell lines are possible candidates for use in the preparation and production of various bioconstructs, especially in cases where the cells of the construct are separated from the internal environment of the body by a barrier, which prevents potential negative effects associated with their use. The main problems in this area are the possible immunogenicity and oncogenicity of cell lines, as well as the decrease in functionality of specific cell lines due to their undergoing the process of immortalization over numerous passages.

## 7. Discussion

Immortalized cells, as well as cell products in general, are increasingly being considered in modern medicine as potential means of therapy for various diseases. This is not surprising: immortalized cells compare favorably with primary cultures because of the possibility of longer passage and their use both in laboratories and clinics. Their use simplifies preclinical and clinical studies of potential drugs because immortalized cells are much more convenient to culture and there is no need for constant isolation of cells from donor material. Compared to primary culture, immortalized cells can be grown in much larger quantities, which is important for large-scale testing. At the same time, immortalized cells are more standardized; when working with them, there is no problem of heterogeneity between donors, which is typical for primary cultures. This facilitates comparisons between different experiments and trials using these cells.

At the same time, certain properties of immortalized cells limit their use for therapeutic purposes. First is the problem of oncogenicity. In almost all cell models, immortalization is achieved by increasing the activity of proteins with proven oncogenic properties: hTERT [26], Myc family proteins [27], viral proteins E6 and E7 of the human papillomavirus [28], and SV40 virus antigens [29]. Therefore, fears about possible tumor growth resulting from transplantation of these cells into the human body are justified, and these fears are probably the biggest barrier to the spread of immortalized cells as a therapeutic agent. Unsurprisingly, in many preclinical studies of these cells, special attention is paid to oncogenicity, which is studied mainly in vitro and using animal models [36,67,106]. However, no study completely excludes the possibility of the cell phenotype transforming into a malignant one. On the other hand, there are various methods of reversible immortalization in which the immortalizing agent—an oncogenic protein—can be “turned off” and cells that do not have oncogenic properties can be used as the final product. However, even reversibly immortalized cells can be dangerous in terms of oncogenicity. For example, women were not included in phase I of the PISCES clinical trial due to the possible activation of c-MycER^TAM^ by tamoxifen, which could be taken by the patients [37]. Another approach is treating the final cellular product to reduce its oncogenic potential, for example, with radioactive radiation, as presented by Sugimoto et al. [59,60]. However, this approach is only applicable to platelets and potentially erythrocytes for the elimination of unwanted precursors. In the field of application of immortalized cells as agents of targeted substance delivery for cancer treatment, this problem is less acute since the presence of high concentrations of anticancer drugs is assumed to be constant in the cell environment; or, when an encapsulation device is used, cells are delimited from recipient tissues and can be removed at any time [78]. Also, many authors are evaluating the possibility of using immortalized cells in conditions where tumor growth is impossible. For example, in the production of an immortalized erythroid progenitor and platelet precursor, in which the final product is nuclear-free under ideal conditions when using filtration systems or other purification methods [54,55,56,57,59,60]; or using immortalized cells as producers of a conditioned medium with therapeutic properties, where there are no cells in the final product when also using filtration systems [89,90,91,93,94]; as well as using immortalized cells as a material for the manufacture of bioconstructions, in particular, a bioartificial kidney and a bioartificial liver, in some variants of the designs in which the cells are delimited from the body by membranes [106,122,123]. Therefore, the oncogenicity of immortalized cells is a serious problem that stands in the way of their application in medicine, so a thorough study of the oncogenic potential of candidate cells is necessary before moving on to clinical trials. On the other hand, the use of reversible immortalization methods can significantly reduce the oncogenic potential of cells, and in some areas of application, the possibility of oncogenic growth is completely absent.

Another concern that attracts the attention of researchers is the change in the properties of immortalized cells due to the immortalization procedure. Several studies have demonstrated that the process of introducing a genetic construct with the gene of an immortalizing agent and subsequent cell selection (if any is performed) can significantly negatively change the properties of the cell population [30,32,33,131]. Considering that the properties of cells are studied more often in primary cultures, one should be careful when transferring the knowledge obtained in these studies to immortalized cells of the same type. An obvious solution to this problem is a direct comparison of the properties of the primary culture and immortalized cells in an experiment; often such a comparison is carried out at the stage of obtaining an immortalized cell line; however, in some studies dedicated to their potential application in practical medicine, the results of such comparisons are also published [69,89,90].

Another important issue is the possible change in the properties of immortalized cells at late passages compared to early passages. It is known that over many passages, chromosomal and epigenetic changes accumulate in cells [31], and such changes affect the properties of cells, including predetermining possible malignant transformation [34]. To our best knowledge, this issue is rarely explored in research on the potential applications of immortalized cells in practical medicine. However, the use of immortalized cells in late passages is assumed in research and practice. Moreover, the possibility of long-term passage is considered one of the main advantages of immortalized cells, which draws attention to them from researchers. We believe that the solution to this issue may be a careful study of the properties of immortalized cells at late passages or the definition of the limit of acceptable numbers of passages for different cell lines.

In this work, we have identified four main areas of practical medicine in which the possibility of using immortalized cells is being actively explored: regeneration, delivery of substances, production of therapeutic factors, and creation of bioartificial organs. Research has advanced the most in the field of the regenerative properties of immortalized cells; the results of several clinical studies with immortalized cells, including phase II, have been published [37,38,41,60,132]. Clinical trials were also carried out with immortalized cells as anticancer drug delivery systems, but only in phase I [78,79]. In these areas, new immortalized lines are being actively developed, and preclinical trials are underway (see summary table, Supplementary Table S1). In the field of using immortalized cells as producers and as a material for bioartificial constructions, the most advanced research is still at the preclinical stage; however, immortalized cells have more potential than primary cells in these areas, so we should expect their use to increase in popularity and move into clinical research.

In almost all areas, immortalized cells have both advantages and disadvantages compared to primary cells, which is reflected in their effectiveness: immortalized cells show both greater and lesser efficacy compared to primary culture (at the same time, research often does not provide this comparison). An exception, in our opinion, is the area of using immortalized cells as producers of therapeutic factors. In this area, immortalized cells have a significant advantage over primary culture, and they have no obvious major drawbacks. In general, immortalized cell lines will be relevant in all areas due to the fact that immortalization solves serious problems associated with the use of primary cells, and, as a result, not only expands the possibilities of existing therapeutic approaches, but also opens up completely new ones.

## 8. Conclusions

Thus, immortalized cells are actively studied in preclinical and clinical studies as potential therapeutic agents for various diseases due to their advantages over primary cell cultures. There are also certain barriers to their application in practice; however, these can be overcome. We believe that immortalized cells have the potential for use in practical medicine, and particularly rapid progress in this direction should be expected in areas where there is at least some concern associated with their oncogenicity.

## Data Availability

Not applicable.

## Supplementary Materials

The supporting information can be downloaded at: https://www.mdpi.com/article/10.3390/ijms241612716/s1.
