# Supplementary material for: Practical Use of Immortalized Cells in Medicine: Current Advances and Future Perspectives
Source: Int J Mol Sci. 2023 Aug 12;24(16):12716. doi: 10.3390/ijms241612716 (PMC10454025; doi:10.3390/ijms241612716)
Supplement: Supplementary file 1 [file ijms-24-12716-s001.zip › ijms-2490040-supplementary.pdf]

Supplementary Table S1: List of cells and approaches mentioned in the review.

Cell line	Cell type	Immortalization method	Immortalization reversability	Use case	Stage	References
Regeneration						
CTX0E03	Neural fetal stem cells	c-MycER^{TAM}	+	Regeneration after ischemic stroke, lower limb ischemia; Alzheimer's disease, Huntington's disease	Preclinical, clinical trials phase I, IIa with published results, IIb without published results	[36–38,41–43,132]
HK532-IGF-1	Neural fetal stem cells	c-Myc linked with estrogen receptor	+	Therapy for Alzheimer's disease	Preclinical in vivo	[47–49]
HB1.F3.BDNF	Neural fetal stem cells	v-Myc	-	Therapy of hemorrhagic stroke, Huntington's disease, spinal cord injury	Preclinical in vivo	[50–53]
HiDEP, HUDEP	Induced pluripotent cells (HiDEP), cord blood cells (HUDEP)	Tat-inducible HPV16 E6/E7	+	Erythroid progenitors are sources of cells for blood replacement procedures.	Establishment	[54]
imERYPC	Embryonic stem cells	Dox-inducible	+		Establishment	[55]

		c-Myc + BCL-XL				
BEL-A	CD34+ bone marrow cells	Tet-inducible HPV16 E6/E7	+		Establishment	[56]
ELLU	CD34+ bone marrow cells	HPV16 E6/E7	-		Establishment	[57]
imMKCL	Immortalized megakaryocytes	Dox-inducible c-MYC, BMI1, BCL-XL	+	Source of platelets	Preclinical in vivo, clinical trial phase I	[59,60]
Different hTERT-immortalized MSCs	Mesenchymal stem cells	hTERT	-	Therapy of the acute phase of ischemic stroke	Preclinical in vivo	[65,66]
YKNK-12	Mesenchymal stem cells	hTERT	-	Bone regeneration with osteoblasts derived from these cells	Preclinical in vivo	[67]
mADSCs^{hTERT}	Mesenchymal stem cells	hTERT	-	Therapy for acute myocardial infarction	Preclinical in vivo	[68]
imMSCs/eSDF-1+	Mesenchymal stem cells	Tet-inducible c-Myc + hTERT	+	Regeneration of peripheral nerves	Preclinical in vivo	[69]
Delivery						

SV40-TK-hfBMSC	Fetal mesenchymal stem cells	SV40 T-antigen	-	Targeted therapy of prostate tumors using cytosine deaminase delivery	Preclinical in vivo	[72]
HB1.F3.CD	Neural stem cells	v-Myc	-	Targeted therapy of various tumors using cytosine deaminase delivery	Preclinical in vivo, clinical trial phase I	[73–77]
IAST/GAL	Rat astrocytes, modified for galanin expression	SV40 T-antigen	_	Therapy of pain syndrome when implantated in the subarachnoid space of the spinal cord	Preclinical in vivo	[80,81]
IAST/hPPE	Rat astrocytes, modified for preproencephalin expression	SV40 T-antigen	-		Preclinical in vivo	[82]
hTERT-BMSCs/Tet-on/GAL	Bone marrow MSCs, modified for galanin expression	Tet-inducible hTERT	+		Preclinical in vivo	[83]
hTERT & CDK4 human myoblasts for encapsulation technology	Human myoblasts modified to produce GM-CSF	hTERT, CDK4	-	Encapsulated GM-CSF-producing cells used for tumor therapy	Preclinical in vivo, planned phase I clinical trial	[86]

Secretome						
Sca-1+/CD31-CSCs^{hTERT}	Resident mouse heart stem cells	hTERT	-	Use of secretome in the treatment of acute myocardial infarction	Preclinical in vivo	[89,90]
SC1$^{GFP/SCX}$	Mesenchymal stem cells	hTERT	-	Use of secretome for muscle tissue regeneration	Preclinical in vitro	[91]
ASC52Telo	Mesenchymal stem cells	hTERT	-	Use of secretome for fibrosis prevention	Preclinical in vitro	[92]
HATMSCs	Mesenchymal stem cells	hTERT	-	Use of secretome for the treatment of chronic ulcers	Preclinical in vitro	[93,94]
S1-ADSC	Mesenchymal stem cells	hTERT	-	Use of secretome for skin regeneration	Preclinical in vitro	[95]
OMLP-PC^{L}	Progenitor cells of the lamina propria of the oral mucosa	hTERT	-	Use of secretome for scar-free tissue regeneration	Preclinical in vivo	[96]
CTX0E03	Neural stem cells	c-MycER^{TAM}	+	Use of the secretome of differentiating cells as a	Preclinical in vivo	[97]

				cardioprotective agent		
Different immortalized MSCs	Mesenchymal stem cells		-	Use of exosomes as a neuroprotective agent, for psoriasis treatment	Preclinical in vivo, phase I clinical trial	[98–101]
Bioartificial organs						
ciPTEC	Epithelial cells of the proximal renal tubules	Temperature-inducible SV40 antigens + hTERT	+	Used in the creation of a bioartificial kidney	Preclinical in vitro	[103,105, 106]
Different immortalized hepatocytes	Adult hepatocytes, fetal hepatocytes	SV40 antigens, native or temperature-inducible, HPV16 E6/E7 + hTERT, Cre/LoxP-controlled hTERT and SV40 antigens	+/-	Used in the creation of a bioartificial liver	Establishment, preclinical in vitro, in vivo	[113–123]
Immortalized keratinocytes	Skin keratinocytes	hTERT	-	Obtaining a model of human skin (use in practical medicine in the original study is not expected)	Preclinical in vitro	[125]

MCEC, hCE-TJ	Corneal endotheliocytes (MCEC), corneal epitheliocytes (hCE-TJ)	SV40 antigens	-	Obtaining models of the cornea (use in practical medicine in original studies is not expected)	Preclinical in vitro	[126,127]
GB/hTERT MSC	Mesenchymal stem cells	hTERT	-	For use in an implantable device that measures glucose levels	Establish ment	[128]
